# Surprising simplicity in the modeling of dynamic granular intrusion

**DOI:** 10.1126/sciadv.abe0631

**Published:** 2021-04-23

**Authors:** Shashank Agarwal, Andras Karsai, Daniel I. Goldman, Ken Kamrin

**Affiliations:** 1 Department of Mechanical Engineering, Massachusetts Institute of Technology, Cambridge, MA 02139, USA.; 2 Department of Physics, Georgia Institute of Technology, Atlanta, GA 30332, USA.

## Abstract

Granular intrusions, such as dynamic impact or wheel locomotion, are complex multiphase phenomena where the grains exhibit solid-like and fluid-like characteristics together with an ejected gas-like phase. Despite decades of modeling efforts, a unified description of the physics in such intrusions is as yet unknown. Here, we show that a continuum model based on the simple notions of frictional flow and tension-free separation describes complex granular intrusions near free surfaces. This model captures dynamics in a variety of experiments including wheel locomotion, plate intrusions, and running legged robots. The model reveals that one static and two dynamic effects primarily give rise to intrusion forces in such scenarios. We merge these effects into a further reduced-order technique (dynamic resistive force theory) for rapid modeling of granular locomotion of arbitrarily shaped intruders. The continuum-motivated strategy we propose for identifying physical mechanisms and corresponding reduced-order relations has potential use for a variety of other materials.

## Teaser

Unexpected simplifications arise in high-speed granular intrusion modeling.

## INTRODUCTION

Intrusions into dry granular media (GM) can create complex flow and force responses, where the media can exhibit both solid-like and fluid-like characteristics. GM deforms elastically under stress like a solid but begins to flow like a fluid once a yield criterion is met. Large variations in the GM’s stress, momentum, and volume fraction in different regions often result in complicated system dynamics exhibiting multiphase characteristics (*1*, *2*). The flow complexity also makes interpreting resistive forces nontrivial if the intruder reinteracts with the deformed region (*3*, *4*), as the GM now has a new inhomogeneous state near the surface. The coupled system of intruder and media becomes challenging to model; the media’s inhomogeneous flow and multiphase nature often restrict modeling to discrete particle methods that track the individual grains, unlike fluids that can be solved with the Navier-Stokes equations.

**Fig. 1:**
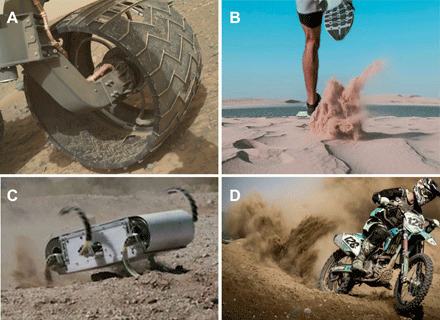
Examples of locomotion on granular surfaces at various speeds. (**A**) Wheel of the Curiosity Mars rover (diameter, **∼**50 cm) (*36*), (**B**) running human (*37*), (**C**) RHex C-legged robot (C-leg limb length, **∼**18 cm) (*38*), and (**D**) a racing dirt bike (diameter, **∼**50 cm) (*39*). Photo credits: (A) MAHLI imager Curiosity, NASA; (B) A. Singh, www.pexels.com; (C) G. C. Haynes, A. M. Johnson, and D. E. Koditschek, University of Pennsylvania; (D) Daniel, www.pexels.com.

A common granular intrusion involves a rigid or flexible solid penetrating into GM and using the resistive force to propel itself into a state of locomotion (see Fig. 1). If a body slowly intrudes into GM, then granular stress arises independent of the intrusion rate, and the resistive force on the intruding body remains in the quasi-static limit (*5*, *6*). However, various intrusion scenarios can arise, which deform the media rapidly enough that the net force response, and hence the locomotive behavior, is affected. Examples of such intrusions include ballistics applications, meteor impacts, rapid locomotion, and many industrial processes (*1*, *7*–*10*).

Rigid wheel locomotion is an exemplar of a system that combines these effects, exhibiting multiphase granular behavior, complex grain-surface interactions, and reinteraction with deformed media. Rigid wheels like those found in planetary rovers (*11*) continuously shear and sometimes rapidly deform the local GM (*12*) to locomote in loosely consolidated terrain. These intrusions, particularly in high-angular-velocity cases, cause the substrate material to behavior to deviate substantially from its quasi-static response, driven by potentially nontrivial surface interactions with the wheel. Thus, we first focus on rigid wheel locomotion as a diagnostic scenario of complex intrusion, which includes a wide array of nontrivial effects.

We propose a continuum framework for intrusion based on a frictional yield condition and free separation. We implement the framework numerically using the material point method (MPM). Our intrusion analysis begins with a focus on driven circular wheels with grousers—grousers are finite-sized radial protrusions along the wheel circumference, which facilitate traction. Grousered wheels are commonly used in granular locomotion applications in soft terrain (*11*–*15*). Alongside scenarios of slow and rapid wheeled locomotion, two additional families of test cases, submerged lateral plate intrusion and “four-flap runners,” are simulated and compared to known results in the literature to verify the model’s ability to capture dynamics of complex granular intrusions. Our proposed continuum model captures the nontrivial rate-dependent phenomena exhibited in complex intrusions although its constitutive equations are rate independent. Our work shows how a single continuum interpretation of GM can represent multiple intrusion scenarios by implicitly reconciling various inertial effects.

**Fig. 2:**
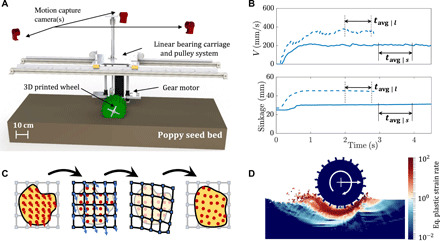
Apparatus for rigid-wheel experiments and continuum simulations. (**A**) CAD model of experimental setup. (**B**) Sample experimental time series data for translation velocity (top) and sinkage (bottom) at low ω (20 RPM, solid lines) and high ω (50 RPM, dotted lines), respectively. *t*_avg∣*s*_ and *t*_avg∣*l*_ show the time windows used for averaging low- and high-ω data, respectively. (**C**) Schematic representation of explicit time integration in a MPM step, whereby a background grid assists in integrating the motion on a set of continuum material points. Solid circles (red) are material points (Lagrangian tracers) and squares (blue) are the nodes of the background mesh. (**D**) A sample continuum simulation using MPM. The field being plotted is the equivalent plastic strain rate.

We also obtain a global-level physical understanding of intrusion dynamics by analyzing plasticity solutions, which guide the development of a reduced-order model for intrusion that we call the dynamic resistive force theory (DRFT). We show that DRFT accurately models all considered granular intrusion cases. By combining existing literature, continuum modeling, and experimental verification, we identify the relevant physics that go into DRFT and its interpretation as corrections to an existing quasi-static resistive force theory (RFT) model (*14*, *16*, *17*) for slow intrusion. Key effects that generate rate-dependent behaviors are identified, and, once incorporated, DRFT allows rapid calculation of the expected resistive forces in GM.

## RESULTS AND DISCUSSION

### Wheel locomotion experiments

Figure 2A shows a Computer Aided Design (CAD) model of the laboratory setup used for performing wheeled locomotion experiments in this study, and Fig. 2B indicates our data collection methodology. More details of the experimental setup are provided in Materials and Methods (and movie S3).

**Fig. 3:**
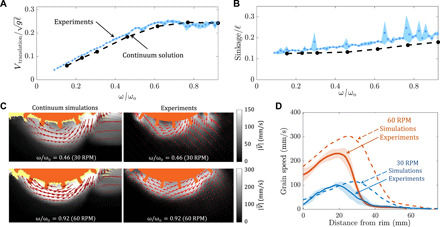
Comparison of wheel locomotion experiments and continuum simulations. Variation of (**A**) translation velocity and (**B**) sinkage from experiments (blue) and continuum modeling solutions (black). The results are nondimensionalized using a characteristic system velocity (*g*ℓ)^0.5^( = 1440 mm/s) for translation velocity; a characteristic system length ℓ( = 212 mm) for sinkage; and a characteristic angular velocity, ω_o_( = (*g*/ℓ)^0.5^ = 65 RPM) for angular velocity, where *g* represents the gravity and l represents the wheel’s outer diameter. (**C**) Granular flow field velocities obtained from continuum modeling and experiments (PIV) for slow (30 RPM, top) and fast (60 RPM, bottom) wheel locomotion. Data are averaged over an effective rotation of 0.1 rad (for PIV), with the orange regions representing the mean position of the wheel. See movies S4 to S6 for more details. The wheel dimensions are provided in Table 1. (**D**) Plots showing variation of grain velocity from continuum simulations and PIV experiments along the radial direction directly below the center of the wheel; note that some wall friction from the plexiglass plate exists in the experiment but not in the continuum solution. Key structural features of the flow under the wheel agree between the experiments and model in (C) and (D).

Figure 3 (A and B) shows the trends of steady-state translation velocity and sinkage (respectively) with increasing angular velocity for a grousered wheel’s free locomotion. Experiments indicate the emergence of a rate-dependent effect in wheel locomotion; an increase in slipping, accompanied by an increase in the sinkage of the wheels, breaks the linear trend in velocity versus ω seen in the quasi-static domain of ω < 30 rotations per minute (RPM) (corresponding to ω/ω_o_ < 0.46 in Fig. 3A).

### Continuum modeling analyses of granular intrusion

We utilize a simple granular continuum model, which captured intruder dynamics in previous studies in the slow, quasi-static regimes (*14*, *18*). Poppy seeds (PS), a model GM used in this study, are modeled as a granular continuum with a Drucker-Prager (rate-independent friction-based) yield criterion, incompressible plastic shear behavior, and a criterion that the material separates into a stress-free media when brought below a critical density. This rheology can be defined by two simultaneous constraints shown below, describing the material’s separation behavior and shear yield condition

$$\text{Free separation}:\;(\rho -\rho_{c})P=0\;\text{and}\;P\geq 0\;\text{and}\;\rho \leq \rho_{c}$$ (1)

$$\text{Frictional yielding}:\;\overset{\cdot }{\gamma }(\tau -\mu_{s}P)=0\;\text{and}\;\overset{\cdot }{\gamma }\geq 0\;\text{and}\;\tau \leq \mu_{s}P$$ (2)

for *i*, *j* = 1,2,3. We define $\sigma_{\mathrm{ij}}^{\prime }=\sigma_{\mathrm{ij}}+P\delta_{\mathrm{ij}}$ as the deviatoric part of the Cauchy stress tensor, *P* = − σ*_ii_*/3 as the hydrostatic pressure, $\tau =\sqrt{\sigma_{\mathrm{ij}}^{\prime }\sigma_{\mathrm{ij}}^{\prime }/2}$ as the equivalent shear stress, μ*_s_* as the bulk friction coefficient, and ρ*_c_* as the critical close-packed granular density. The (plastic) flow rate tensor is *D_ij_* = (∂*_i_v_j_* + ∂*_j_v_i_*)/2 and $\overset{\cdot }{\gamma }=\sqrt{2D_{\mathrm{ij}}D_{\mathrm{ij}}}$ is the equivalent shear rate. When shearing plastically, the stress and flow rate are presumed to align (e.g., $\sigma_{\mathrm{ij}}^{\prime }/2\tau =D_{\mathrm{ij}}/\overset{\cdot }{\gamma }$). The model evolves the flow by solving the momentum balance equations, $\partial_{j}\sigma_{\mathrm{ij}}+\rho g_{i}=\rho {\overset{\cdot }{v}}_{i}$. Below the yield criterion, the grains act like a linear-elastic solid, so that our model is elastic plastic in the dense regime. We assume that a constant surface friction coefficient describes the interaction of the granular continuum with solid-body surfaces. The media density, material internal friction, media-surface friction and other material inputs are included in table S1 (in the Supplementary Materials).

We use the MPM algorithm described in Dunatunga and Kamrin (*19*, *20*) to implement these constitutive equations assuming two-dimensional (2D) plane-strain motion. A schematic representation of an explicit time integration MPM step is shown in Fig. 2C; the material points carry the continuum data and are moved each step with the help of a background grid (more details in Materials and Methods). Figure 2D shows a sample wheel locomotion using MPM, plotting the variation of equivalent plastic shear rate in the system.

The trends of steady-state translation velocity and sinkage with varying ω obtained using continuum modeling are plotted in Fig. 3 (A and B). Continuum modeling successfully captures the experimental trends for wheel locomotion; in particular, the model captures the plateau in the normalized *v* − ω curve at the correct rotation speed and correctly predicts increased sinkage with rotation rate.

To check robustness of the results, we also applied small changes to the initial state of the experimental and simulated systems, including variations in initial wheel depth, initial wheel velocity, and ramp rate of the wheel, and observed that the steady-state results were insensitive to these variations (*21*).

To further validate the model predictions, we have conducted experiments to visualize subsurface flow fields and compared them to the model. The experiments place the wheel adjacent to a clear plexiglass plate so a camera can capture the underlying grain motion with particle image velocimetry (PIV) (*6*). Velocity fields in grains for 30 and 60 RPM cases from continuum modeling and experimental PIV analysis are plotted in Fig. 3 (C and D). We posit that wall drag from the plexiglass plate likely causes the granular flows in the experiment to be overall slower than the model; however, the key structural features of the flow under the wheel agree between the experiments and model. Both show a zone of material ahead of the wheel being pushed forward and a wide zone under and behind the wheel being pushed to the rear. The rear flow zone also grows with increasing ω due to higher flow entrainment and material movement at higher ω.

### Toward reduced-order models

A major benefit in identifying an accurate continuum model for a system is the possibility of using it to extract global-scale simplifications of the system’s dynamics that can be used to develop further-reduced models. For example, in previous work on slow quasi-static intrusion, Askari and Kamrin (*18*) found a connection between frictional yielding and a reduced-order intrusion force model called granular RFT (*16*). The success of the present continuum model for slow and rapid locomotion in wheels (and the other intrusion scenarios in this study) motivates us to ask whether an RFT-like reduced-order model for complex, rapid intrusions exists and if it might be derivable based on phenomena observed within the continuum model. We begin by first defining the quasi-static form of RFT and evaluating its predictions for wheeled locomotion dynamics.

RFT is an empirical methodology that has been successful in estimating the force response for arbitrarily shaped intruding geometries in the quasi-static limit, permitting direct simulation of locomotion in granular volumes (*14*, *16*, *17*). RFT assumes the stress on a small surface element of an intruder follows a localized formula in which depends only on the motion, location, and orientation of that element (*22*). This local formula decouples the stress response among the surface elements of an intruder, thereby permitting RFT to predict intrusion forces with near real time numerical calculations.

In a coordinate system where *z* points positive upward with granular free surface at *z* = 0, and *x* is a chosen horizontal axis perpendicular to *z*, RFT presumes the force-per-area vector (or traction) **t**, on each surface element can be written as **t** = **α**(β, γ) *H*( −*z*)∣*z*∣, dependent on the element’s orientation angle (β), velocity angle (γ), and vertical depth from the free surface (∣*z*∣), with *H* being the Heaviside function. The empirical traction-per-depth vector **α**(β, γ) = (α*_x_*(β, γ), α*_z_*(β, γ)) is measured with small plate intrusion experiments, which vary β and γ. By summing these locally defined tractions, RFT predicts the net resistive force and moment on the entire intruder surface *S*. For example, RFT gives the following intrusion force formula

$$F=\int_{S}\alpha (\beta ,\gamma )\;H(-z)|z|\;\text{dA}$$ (3)

**Fig. 4:**
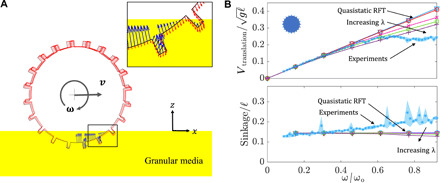
Experiments versus RFT. (**A**) Snapshot of a quasi-static RFT simulation, used for studying grousered wheel locomotion. Direction and magnitude (normalized) of the velocity and resistive stress are indicated by red and blue arrows, respectively, along surface elements of the wheel boundary. (**B**) Translational velocity (top) and sinkage behaviors (bottom) of the wheel; experimental mean and 1σ SD (light blue data) and RFT results with local λρ*v*^2^ modification (solid lines). The results in (B) are nondimensionalized as explained in Fig. 3. The direction of increasing λ is indicated (λ = 0, 1, 25, 50, and 100). Red solid lines with λ = 0 [in (B)] correspond to quasi-static RFT results.

Figure 4 (A and B) shows the results of applying quasi-static RFT (solid blue line in Fig. 4, A and B) in modeling grousered wheel locomotion. In implementing the RFT model of locomotion, we also use a “leading edge hypothesis” to ensure that resistive forces experienced by the wheel consist of contributions only from surface elements that move “into” the sand, i.e., surfaces whose outward normal (***n***) and velocity (***v***) make a positive inner product (***n*** · ***v*** > 0). We use the established RFT functions α*_x_* and α*_z_*, for the GM used in our experiments (*16*). Figure 4B shows that while RFT captures the speed versus ω trends at low ω, at higher ω it does not predict the wheel locomotion kinematics. RFT predicts a linear relation between the angular and translation velocities, which matches the experiments’ dynamics at low speeds, but diverges as ω increases. The fact that quasi-static RFT predicts the steady speed of a round wheel to always be a constant multiple of the wheel spin can be shown as a consequence of the rate independence of the RFT traction relation in Eq. 3 (see section S2 for more details).

### Exploiting the continuum treatment for physical insight

An important step in developing a general reduced-order model for high-speed granular intrusion scenarios is to identify the key underlying physics. In granular intrusions, rate effects could arise due to a variety of physical causes. Increased vibrations in the media could fluidize the material at high speeds and reduce its strength (*23*). Increasing velocities could decrease the friction on the wheel/media interface (per a dynamic friction drop), which, in turn, could decrease the traction on the wheels. Rapid flows may also have substantial micro-inertia, which makes the rheology rate dependent by causing the stress ratio μ ≡ τ/*P* to depend on shear rate through the “inertial number” *I*, where $I=\overset{\cdot }{\gamma }\sqrt{d^{2}\rho_{s}/P}$, where $\overset{\cdot }{\gamma }$ is the shear rate, *d* the mean grain diameter, ρ_s_ the solid particle density, and *P* the local pressure (*5*). Moreover, conventional macro-inertia (i.e., the $\rho {\overset{\cdot }{v}}_{i}$ term in the momentum balance equation) adds inertial body forces that could alter the flow of the media and its resistance against the intruder.

Predicting the dominating rate effect(s) is difficult using experiments alone. In this regard, our continuum modeling approach greatly aids in eliminating candidates from the possible rate effects above. The key is to recall that our model implements a rate-insensitive frictional surface interaction with no dynamic friction drop on the wheel-sand interface and a rate-insensitive constitutive model with no dependence on the inertial number nor any accounting of material thermalization or fluidization. The model does, however, include macro-inertia in the momentum balance equations. The fact that the continuum model is successful in capturing the wheel dynamics along with many other granular intrusion scenarios (discussed later) indicates that the observed rate effects should be reconcilable solely from macro-inertia ($\rho {\overset{\cdot }{v}}_{i}$). At the same time, the global consequences of local macro-inertial forces may be subtle and depend on the particular system and its dynamics.

On the basis of this insight, along with analysis of the continuum solutions to wheel locomotion and other granular intrusion scenarios from the literature, we now propose and test a more general RFT that encompasses the domain of slow to rapid intrusions in GM, which we refer to as DRFT.

### Dynamic resistive force theory

DRFT modifies the quasi-static RFT in two ways to account for macro-inertial effects. First, we add a momentum flux contribution, which we term the dynamic inertial correction. This term is required for the transfer of momentum to the granular material surrounding the intruder. This term is also in accord with many previous studies on high-speed granular intrusions (*24*–*30*) and takes the form of an additional rate-dependent force going as velocity squared. The second modification, which we will show is critical for more complex intrusions, describes the way in which increased bulk inertia can change the free-surface geometry. A change to the free-surface geometry then feeds back on the resistive forces through the depth dependence of RFT. We denote this modification as the dynamic structural correction. Together, DRFT imposes the following formula for the traction on a surface element

$$t=\alpha (\beta ,\gamma )H(-\tilde{z})|\tilde{z}|-n\lambda \rho v_{n}^{2}$$ (4)

where $|\tilde{z}|$ indicates the effective depth of the surface element. That is, $\tilde{z}=z+\delta h$ where δ*h* represents the height decrease of the free surface in the zone affecting the traction at (*x*, *z*). Recall **n** represents the outward normal to the surface element (and −**n** the inward), and we define *v*_n_ as the normal component of the surface velocity. To use DRFT, one must determine the appropriate δ*h* for each surface element of the intruder as a function of the intruder motion and an appropriate λ, an *O*(1) scalar fitting constant. Similar to RFT, DRFT asserts a localized formula for the calculation of stresses on intruder subsurfaces and thus allows for near real-time modeling of intruder motion.

### Understanding the dynamic inertial correction

We take a moment to discuss the two dynamic corrections included in DRFT, beginning with the dynamic inertial correction. Analysis of the momentum balance equations under certain simplifying circumstances (see section S1) allows one to deduce that the transition from a quasi-static flow to a faster flow comes with a resistive force increases as $\rho {\mathrm{Av}}_{n}^{2}$, similar to dynamic pressure in a fluid, where *A* is the intruder area. Physically, this term represents the reaction force that comes from transferring momentum to the GM.

A number of previous studies (*24*–*30*) have modeled the rate dependence of intrusion force similarly, by adding a term proportional to normal speed squared to a depth-dependent “static” term. Examination of experimental data in (*26*, *31*) agrees with a rate-dependent force addition of the form $\lambda \rho {\mathrm{Av}}_{n}^{2}$ in simple vertical and horizontal intrusions (see figs. S2 and S3 and movies S1 and S2), where λ is a *O*(1) scalar fitting constant that accounts for certain approximations in the analysis (see section S1).

It is natural to ask whether the addition of a velocity-squared term to the quasi-static RFT relation is enough alone to explain the rate dependence observed in general intrusion scenarios, including wheeled locomotion. We suppose the surface traction is modeled to obey the relation in Eq. 5 below and use this relation to re-evaluate the grousered wheeled locomotion problem

$$t=\alpha (\beta ,\gamma )H(-z)|z|-n\lambda \rho v_{n}^{2}$$ (5)

Figure 4B shows the results for various values of λ. The case of λ = 0 represents the previously discussed quasi-static RFT in these graphs. The introduction of the inertial force term (λ > 0) adds a new force contribution having net force components upward and opposite to the horizontal direction of wheel translation. This upward force results in a decrease in wheel sinkage, opposite to the experimental observation. The magnitude of these extra forces is very small; the prefactor λ was varied from 1 to 100 in an attempt to match the experiments, but this has little effect on the outcome and the trends for both velocity and sinkage cannot be matched (Fig. 4B). It is clear that the dynamic inertial correction alone is not sufficient to describe this set of tests.

### Understanding the dynamic structural correction

**Fig. 5:**
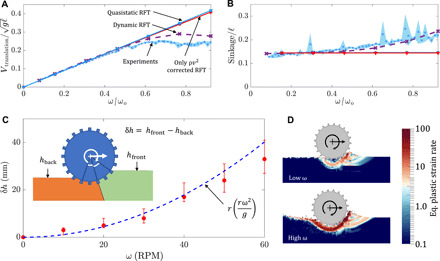
Dynamic RFT captures experiments and continuum modeling. Variation of (**A**) wheel translation velocity and (**B**) sinkage from experiments compared to quasi-static RFT, DRFT, and DRFT without any dynamic structural correction (i.e., having only the ∼ρ*v*^2^ correction). (**C**) Presumed zones of influence and effective free-surface variation for constructing the dynamic structural correction; δ*h* represents the gap between effective front and back free-surface positions. MPM data (red circles) and empirical fit (blue dotted line) for δ*h*. (**D**) Variation of equivalent plastic strain rate magnitude obtained using MPM continuum modeling for slow-speed (30 RPM) and high-speed (90 RPM) wheel locomotion. See movie S7 for visualizing the variation over time. The results in (A) and (B) are nondimensionalized as explained in Fig. 3.

To understand the rationale behind the dynamic structural correction in DRFT, we start by considering the spatial variation of plastic strain rate magnitudes from continuum modeling simulations for low and high ω cases shown in Fig. 5D. The plots make it possible to visualize how different portions of the wheel derive their resistive forces from different zones of the GM. While the strain rate profiles change as angular velocities increase, the basic patterns of shearing remain similar. The sheared material reaches the free surface of the granular volume in two zones. Approximately half of the flow originating from the leading edge of the wheel reaches the free surface on the trailing rear face of the wheel. The remaining flow lines extend to the free surface on the leading front face of the wheel. The height of the free surface on the rear side of the wheel decreases with increasing ω; qualitatively, as ω grows, the wheel expels material on the rear side. The reduction in rear free-surface height suggests a reduction in the pressure head and consequent weakening of the material in the rear shear zone. This is the key observation which motivates the form of the dynamic structural correction.

Figure 5C shows the free-surface height reduction, δ*h*, as measured from the continuum model simulations by identifying the lowest point making rear contact with the wheel for which hydrostatic pressure →0. The more rapidly the wheel spins, the deeper this point descends. Given the paucity of parameters in the continuum model, dimensional analysis is useful; for a given substrate material, it suggests the form δ*h* = *r* · ψ(*r*ω^2^/*g*) for some function ψ. Unexpectedly, we find that ψ is well approximated by the identity function. The fit of δ*h* = *r* (*r*ω^2^/*g*) and the continuum modeling results in Fig. 5C show good agreement. Combined with the understanding developed in the previous section, the form of the effective free surface is approximated using a simple partition as shown in Fig. 5C, with the rear zone of the wheel set to have a constant free-surface height reduction *h*_back_ differing from the initial free-surface height (undisturbed medium height) by a term δ*h* = *r* (*r*ω^2^/*g*). To select the dividing angle delineating the front- and rear-affected zones of flow, we choose to equally divide the contact zone for driven wheels. Our choice is driven by the simplicity of this division, also observing a similar division of contact zones for representing traction on wheels by Hambleton and Drescher (*32*). This new model changes the effective free-surface heights only for the surface elements closer to the rear of the intruding wheel surface.

By including this effective free-surface height formulation, we now arrive at DRFT, Eq. 4. We implement this DRFT model using the same implicit RFT code framework discussed in Materials and Methods, using λ = 1 and ρ ≈ ρ_c_ = 638 kg/m^3^. The trends of translation velocity and sinkage with respect to ω now show good agreement between experiment and DRFT (Fig. 5, A and B). We also include, for comparison, what the solution is when only the dynamic inertial correction is used. While DRFT combines both dynamic corrections, it is clear that the dynamic structural correction dominates the dynamic inertial correction in the case of wheeled locomotion. While we have presumed for simplicity that the division between the two contact zones takes place halfway through the wheel-sand interface, it can be seen in Figs. 3C and 5D that the division may actually be closer to the front of the wheel. This could explain our slight overprediction of speed for high ω (Fig. 5A). A second set of grousered wheels tests involving a smaller wheel are included in the Supplementary Materials (see fig. S1 and section S3), and DRFT works equally well without the need to refit the function for ψ used for δ*h*.

The agreement with DRFT suggests that the low-to-high slip transition in wheeled locomotion (where slip= 1 − *v*/*r*ω for *v* is the translational velocity, *r* is the nominal radius, and ω is the angular velocity of the wheel) occurs largely because more rapidly spinning wheels remove material from behind the wheel, which reduces the pressure in the rear zone, thereby weakening the base of material that would otherwise provide a scaffold off of which the wheel pushes. Updating RFT by accounting for this effect has appropriately captured the dynamics of the complex wheel locomotion scenario in a reduced-order modeling framework.

### Additional verification studies for the continuum model and DRFT

The wheel tests provide a complex intrusion scenario and have a dynamic structural correction that is much larger than the inertial correction. To check the robustness of our continuum modeling approach and Eq. 4 for DRFT, we now examine the converse situation with two additional sets of simulations—submerged plate intruders and locomoting runners. We evaluate these cases based on data from continuum solutions, validations against the literature, and the arguments in the previous section and expect the dynamic structural correction to be small and the dynamic inertial correction to dominate. Visually, these cases represent two separate classes of intruders. While the dragged plates represent forced motion, the runners represent a class of self-propelling locomotors that may appear similar to the prior studied wheels. Yet, force responses in both cases are dominated by the dynamic inertial correction (more details in the following sections) and do not mimic behavior of the grousered wheel. Thus, these distinct cases test the breadth of the modeling capability of DRFT.

#### Submerged horizontal intrusion

**Fig. 6:**
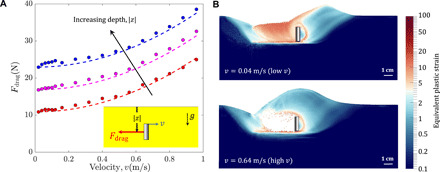
Modeling slow-to-rapid plate intrusion. (**A**) Continuum MPM data (colored circles) and *K*∣*z*∣+ λρ*Av*^2^ fits (dotted lines) for horizontal intrusions at various depths (∣*z*∣) ([20,30,40] mm), where *K* = 580 N/m and λ = 1.1. (**B**) Variation of equivalent plastic strain rate for (top) low-velocity (0.04 m/s) and (bottom) high-velocity (0.64 m/s) intrusion cases (at 30 mm depth). See movie S2 for the video. Simulations are plane strain.

Thin plates submerged in a GM at various fixed depths (20 to 40 mm) are dragged horizontally at different speeds using continuum modeling. The continuum model runs in plane strain, where the plate has a length of 0.016 m and the effective medium density is 900 kg/m^2^. The chosen density is similar to that of ground coal or marble. The filled circles in Fig. 6A show the observed drag force variations with the drag speed. Experimental studies by Schiebel *et al.* (*31*) found the variation of drag forces in such a scenario to follow the trend *K*∣*z*∣+ λρ*Av*^2^ (see fig. S1), where *K* and λ are constants, ∣*z*∣ is the depth of the plate below the free surface, ρ is the effective granular density, *A* is plate area, and *v* is horizontal plate velocity. Our continuum modeling also obtains the same trend (Fig. 6A). In the slowest cases (*v* ∼ 0), we obtain a linear force versus depth relation, *F*_drag_ = *K*∣*z*∣ for *K* = 580 N/m. As speed increases, we find that continuum predictions match the data well at three different depths, for λ = 1.1. Incidentally, the same value of λ also matches the rate dependence observed in the Schiebel *et al.* (*31*) experiments for horizontally driven intruders at the free surface.

A comprehensive understanding of the resultant form of the drag force trends can be obtained by observing continuum modeling results in the context of DRFT. Figure 6B shows the deformation profiles around the plate at two selected speeds (which differ by about an order of magnitude). The profiles in Fig. 6B of high- and low-speed intrusion suggest that the intruder tractions arise from pressing the granular material in front of the plate upward and to the right, toward a common free-surface height, *h*_front_. The rear flow zone, which changes in slow versus high-speed intrusion, is either in the separated phase or newly consolidated as it falls and fills in the gap behind the moving plate. Likewise, the rear media makes a negligible contribution to the resistive plate force; no part of the of the rear face of the plate is a “leading edge” satisfying ***n*** · ***v*** > 0, so forces approximately vanish there. This is in contrast to the grousered wheel case, where, due to rotation, a substantial portion of the back half of the wheel is a leading edge that can interact nontrivially with media behind the wheel. We thus expect a negligible dynamic structural correction for horizontal plate drag, due to the lack of leading edge on the rear face of the plate and an approximately speed-independent *h*_front_. Indeed, the force relation *F*_drag_ = *K*∣*z*∣+ λρ*Av*^2^ obtained from experiments as well as continuum modeling, displays only the dynamic inertial correction of DRFT as expected. These results are in accord with our hypothesis and confirm the DRFT prediction for submerged horizontal intrusion. For similar reasons as just discussed, we expect symmetric vertical intrusion of plates to also invoke a negligible structural correction; see the Supplementary Materials (fig. S3) for details and confirmation against DRFT. Note that in our plate drag studies, we have restricted our intrusion depths to within an *O*(1) factor of the plate width. This depth range indicates the approximate limits of RFT, as beyond such depths, the assumptions of RFT (such as a linear dependence of granular resistance with depth) begin to degrade (*33*).

#### Four-flap runner

While the dragged plates are forced to move at set speeds, we also study a self-propelling locomotor, a four-flap runner, whose locomotion speed is determined via the interactions of the locomotor’s self-actuated limbs (flap motion) and the substrate dynamics (geometric details are in table S1). The low number of flaps, along with the large flap length to inner radius ratio, minimizes the interaction between neighboring flap intrusions of the runner’s resultant granular flow.

The runner takes inspiration from the experiments of Li *et al.* (*16*) and Zhang *et al.* (*34*) with running C-legged robots (similar to Fig. 1C). Li *et al.* (*16*) drove their robots with dimensionless spin ratios (ω/ω_o_) ranging from 0 to 1.25 ((ω_max_, ω_o_) = (240,190) RPM) and observed a decreasing slip with increasing angular velocity in their experiments. Similarly, Zhang *et al.* (*34*) tested locomotion over a larger ω/ω_o_ range of 0 to 3.8 ((ω_max_, ω*_o_*) = (720,190) RPM) and observed that, in the higher range of spins, the sinkage in their experiments breaks away from trends observed by Li *et al.* (*16*), i.e., robots elevate above their resting depth. Their running robots display qualitatively opposite behaviors to grousered wheels: As rotation rates increases, runners sink less and move faster, whereas wheels sink more and travel slower. We explore whether the fundamental physics of such qualitatively reversed behavior is already embedded in our continuum modeling and consequent DRFT framework. Because our current continuum modeling capabilities were limited to planestrain (2D) problems, we cannot implement a full C-legged robot running in 3D. We take the four-flap runner as a representative of the family of runners and explore our 2D continuum model’s capability in modeling such behaviors.

**Fig. 7:**
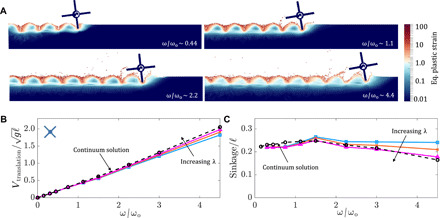
Running on GM. (**A**) Continuum model solutions for the equivalent plastic strain at increasing angular velocities ω for four-flap runner locomotion (ω_o_ = 65 RPM). See movie S8. Continuum solutions from MPM (black dotted line with “o” markers) and DRFT solutions (solid lines) for translational velocity (**B**) and sinkage (**C**) versus angular velocity, ω, in four-flap runner locomotion. DRFT solutions for λ = 0, 2, and 4 pictured. The results in (B) and (C) are nondimensionalized as explained in Fig. 3 with ℓ = 190 mm (runner’s outer diameter).

In the continuum modeling, the dimensionless mass ratio of the runner, given by *m*/ρ_c_𝓁^2^*W* for *W* the out-of-plane width, is set to be in the same range (≈6) as the corresponding 20 grousered wheels shown previously to keep the comparison between runners and grousered wheels relevant. For similar reasons, we keep the runner diameter similar to that of grousered wheel (190 mm versus 212 mm). The angular velocity of the runner is varied over a range of 10 to 300 RPM, which corresponds to a dimensionless spin ratio range varying from ω/ω_o_ = 0 to 4.5 (ω_o_ = 65 RPM). The continuum results (see Fig. 7, B and C) show qualitative agreement with the findings of Li *et al.* (*16*) and Zhang *et al.* (*34*)—with increasing spin rate, a decrease in effective slip and an elevation of the wheel above the rest depth is observed. Incidentally, the turnover in elevation for our runners was found at a spin ratio ∼1.4, similar to that obtained by Zhang *et al.* (*34*).

We now use the continuum model as a baseline reference to evaluate the DRFT performance for runners. Figure 7A shows the variation of the equivalent plastic strain for four different angular velocities in the continuum model. As expected, because of the relatively large separation between intruding legs, there is no visible self-interaction of the granular material between intrusions and the free-surface height directly behind intruding legs remains unchanged, which suggests a minimal role of the dynamic structural correction. This observation guides us to model these scenarios using DRFT with typical, *O*(1), λ values (λ = 0,2,4) and no dynamic structural correction. Figure 7 (B and C) shows the resulting steady-state sinkage and translation velocity at various angular velocities from DRFT calculations (solid lines). DRFT appears to capture the kinematic trends of the reference solution, approaching quantitative accuracy for λ ∼ 4. With this result, it is encouraging to note that DRFT has captures the dependence on ω in both runners and grousered wheels, which behave in opposite ways as ω increases.

Our four-flap runner study also explains the observations of the above-mentioned C-legged robot studies. We believe that the quasi-static RFT modeling in Li *et al.* (*16*) was sufficient because the dynamic inertial correction was still small in their tested range (in our study, the dynamic inertial correction becomes noticeable only above ω/ω_o_ ratio of ∼1.2). Zhang *et al.* (*34*) go to higher rotation rates, revealing the nontrivial elevation and slip trends due to rate that we see in continuum and DRFT solutions.

### Conclusion

In this work, we have focused on evaluating the effectiveness and implications of a continuum model for problems of granular intrusion up to high speeds, which allows for detailed modeling of complex multiphase inhomogeneous granular systems. We have observed two unexpected results. First, a continuum model based only on a constant friction coefficient and tension-free separation is able to quantitatively model complex granular intrusions in a variety of scenarios. Second, we find that just two macro-inertial corrections to RFT allows successful modeling of granular intrusions across speed regimes.

These results were obtained progressively. By analyzing the continuum model’s solutions, an understanding of the key physics involved in such complex intrusion scenarios was identified, which, in turn, motivated the ingredients of DRFT. DRFT allows for robust, near real-time modeling of granular intrusion in a large variety of cases, including self-propulsion. Our study of rigid intrusion into GM indicates that the force response upon intrusion consists of two primary rate-dependent modifications: (i) a dynamic inertial correction and (ii) a dynamic structural correction. The dynamic inertial correction accounts for the momentum transfer to the surrounding material, whereas the dynamic structural correction describes how a rapidly moving intruder can change the pressure head by modifying the free surface. Both effects are related to the macro-inertia of the media (stemming from the $\rho {\overset{\cdot }{v}}_{i}$ term in the momentum balance partial differential equation). For the scenarios considered here, micro-inertial effects [per a μ(*I*) rheology] are not significant even though the motion appears “fast”—previous work on rapid projectile penetration (*20*) indicates that the high pressures that develop around rapid intruders tend to keep *I* relatively small. Hence, we have reached the rather interesting conclusion that the observed rate-dependent dynamics are reconcilable with a rheology that is rate-independent. In terms of limitations, it is known that quasi-static RFT loses accuracy when intruders are too deep, as the linear force versus depth dependence eventually plateaus in the lift direction (*33*) for slow intruders. We expect the same constraints on depth to apply to DRFT as well.

Dynamic RFT has enough generality to explain two opposing scenarios: weakening of the GM during grousered wheel locomotion, as well as strengthening of the GM during rapid running. We have shown that DRFT accurately predicts the GM system behaviors in the limiting cases, i.e., when one of the two dynamic effects is dominant. Further studies are required to fully test the model for mixed cases where both dynamic corrections are significant. We have assumed additivity, in line with previous notions of a static component and an inertial component of the intrusion force (*6*, *23*, *25*, *30*). However, it is possible that a more complicated functional combination may arise.

Although our study has mainly focused on dry noncohesive GM, the formulation of DRFT in granular flows suggests the existence of other similar reduced-order models in other materials. A combination of experiments and continuum modeling proved vital in this study for verifying the underlying physics. The proposed continuum framework can easily be modified to encompass a large variety of materials once their constitutive equations are known. Future work may explore faster methods of predicting flows, along with various complex intruders to systematically determine the form of the dynamic structural correction. Further studies could also explore the existence of similar reduced-order models for related classes of materials like noncritical state GM, cohesive sands/muds, and fluid-saturated sands.

## MATERIALS AND METHODS

### Experiments (wheel locomotion)

To perform systematic experiments of free-wheel locomotion, we built a automated “terramechanics testbed.” A powerful gear motor (capable of providing up to 70 RPM at 14.1 Nm) is mounted in a carriage (Fig. 2A), which moves freely along vertical and horizontal linear bearings. We control the effective vertical loading of the wheels through a combination of weights and pulleys. The system runs trials in a fluidizing bed of Poppy Seeds (a dry noncohesive GM) across a bed length of 1 m, allowing for controlled resets of terrain by blowing air up from the bottom. The PS act as the representative material for the class of noncohesive granular materials in our study. We specifically choose them due to the ease of running wheel locomotion experiments within them and previous experience using RFT. This fluidization redistributes the grains evenly into a homogeneous medium after each experiment, giving nearly identical terrain for each test (*35*). Along with the terrain fluidization, the testbed also has the capability to reset itself: After each run, a linear actuator and a winch work together to drag the wheel carriage back to its starting position. Various system dimensions/specifications are listed in table S1.

For experimental visualization of the granular flow around the wheels (Fig. 3C), we also perform PIV analysis of the wheel locomotion at different ω values. We place the wheel adjacent to the transparent side wall of the Poppy Seed container and perform the locomotion trials. Images of the flow field are captured with a high-speed camera mounted on a tripod at a resolution of 1280 × 1024 and a framerate of 500 frames per second. We expect minor variations in the flow fields due to the friction experienced by the material flowing next to the sidewall. The open-source PIVLab package was used in MATLAB for the analysis.

### RFT modeling

We implement RFT simulations using independent experimental variables and an implicit iterative scheme. A sample simulation diagram is shown in Fig. 4A. Using the rigid wheel assumption, the wheel surfaces are discretized into smaller subelements, which, as a whole, approximate the total geometry. The orientation angle (β), velocity angle (γ), effective depth from the free surface (∣*z*∣), and area (*dA*) of each subsurface are used along with RFT assumptions of locality and additivity of granular resistive forces and a leading edge hypothesis (discussed earlier) to find net the resistive force and moment. In doing so, Eq. 3 is evaluated using established RFT coefficients from (*16*) and the associated scaling coefficients from table S1. A momentum balance in the *x* and *z* coordinates then models wheel motion in the horizontal and vertical direction. The effective heights of wheel grousers are also taken to be one-third of their true physical length (based on experimental PIV data) to account for the shadowing effect (*15*). Convergence studies of the force response determine the discretization fineness of the wheel shape. Each inner-circumferential subsurface lug is divided into 14 elements, and each of the lug surfaces (1 normal and 2 side-wise) was divided into 8 elements. Thus, the wheel has 570 surface elements in total. For the DRFT implementation, only the effective heights experienced by surface elements on the rear side of the wheel were modified. This height modification was based on the formulation shown in Fig. 5C. The rear region was taken as the rear half of the contact area between sand and wheel (see Fig. 5D). The division was based on the angle subtended by the contact region at the wheel center.

### Continuum modeling

We use Material Point Method to carry out the continuum modeling of the system. In MPM, material is discretized as a set of material point tracers that carry the full continuum state. Data from these tracers, representing a small volume of material around their position, are cast onto a background simulation grid where the equations of motion are solved. Thus, material point tracers act as quadrature points for solving the weak form of the momentum balance equations on a static background simulation grid. A forward-Euler time integration method was used to update the material position and properties. A representative schematic of a time step update is given in Fig. 2C. We model the wheel as a high-stiffness elastic solid with a fixed angular velocity, which is instantaneously enforced on the wheel. In terms of simulation resolution, we use a 200 × 200 grid representing a domain size of 1 m × 1 m with initial seeding of 2 × 2 material points per grid cell.

## SUPPLEMENTARY MATERIALS

Supplementary material for this article is available at http://advances.sciencemag.org/cgi/content/full/7/17/eabe0631/DC1
